# Effects of miRNA-199a-5p on cell proliferation and apoptosis of uterine leiomyoma by targeting MED12

**DOI:** 10.1515/med-2021-0348

**Published:** 2022-01-10

**Authors:** Wei Zhao, Yingyan Zhao, Ling Chen, Yan Sun, Sumei Fan

**Affiliations:** Department of Clinical Laboratory, Women’s Hospital of Nanjing Medical University, Nanjing Maternity and Child Health Care Hospital, Nanjing, Jiangsu 210004, China; Department of Obstetrics and Gynecology, Zhangjiagang Hospital of Traditional Chinese Medicine and Affiliated Zhangjiagang Hospital of Nanjing University of Chinese Medicine, Zhangjiagang 215600, China; Department of Geriatrics, The Affiliated Huai’an Hospital of Xuzhou Medical University and The Second People’s Hospital of Huai’an, No. 62, Huaihai Road (S.), Huaian, Jiangsu 223002, China

**Keywords:** uterine leiomyoma, MiR-199a-5p, MED12, proliferation, apoptosis

## Abstract

**Background/aims:**

Uterine leiomyoma (ULM) is a kind of gene-involved benign tumor, which is located in the front of female reproductive tract. It is one of the most common reproductive tract tumors in women, which leads to abnormal menstruation, repeated pregnancy loss, and other serious gynecological diseases. Recently, microRNAs (miRNAs) have attracted much more attention in the process of exploring the molecular mechanisms of tumorigenesis. Furthermore, the deregulated miRNAs had been reported to play important roles in ULM pathology.

**Methods:**

In this study, we assessed the expression level of microRNA-199a-5p (miR-199a-5p) in human ULM by quantitative polymerase chain reaction. After that cell counting kit 8, colony formation, 5-ethynyl-20-deoxyuridine, flow cytometry, and Western blot analyses were performed to investigate the effects of miR-199a-5p on ULM cell proliferation and apoptosis.

**Results:**

We confirmed that miR-199a-5p was significantly downregulated in human ULM. The results of function analyses showed that miR-199a-5p inhibited cell proliferation and induced cell apoptosis *in vitro*. Bioinformatics tool showed oncogene *MED12* was one of the target genes of miR-199a-5p, which mediated the effect of miR-199a-5p on the ULM.

**Conclusion:**

Our results showed that miR-199a-5p functioned as an antitumor factor in human ULM cells. These findings broaden the current findings on the function of miR-199a-5p into the ULM pathogenesis, and miR-199a-5p may serve as a prognosis and therapeutic target for the ULM and its related diseases.

## 1 Introduction

Uterine leiomyoma (ULM), a kind of gene-involved benign tumor, is located in the front of female reproductive tract. It is one of the most common reproductive tract tumors in women, leading to abnormal menstruation, repeated pregnancy loss, and other serious gynecological diseases [1,2,3]. Besides, this disease has serious negative impacts on the quality of life and financial burden [4,5,6]. However, due to the ambiguous understanding of ULM molecular pathogenesis, the definite etiology of ULM is still elusive. Thus, finding a new and effective potential target for treating ULM is of great urgency.

MicroRNAs (miRNAs) are a kind of small noncoding RNA that is 22–24 nucleotides in length [7]. By binding to the complementary sequences in the 3′-untranslated region (3′-UTR) of mRNAs, they repress gene expression levels and prevent their translation [8]. Recently, miRNAs have attracted much more attention in the process of exploring the molecular mechanisms of tumorigenesis. The deregulated miRNAs have been reported to play important roles in ULM pathobiology [9,10,11]. For instance, miRNA-197 was found to be significantly downregulated in human ULM and regulates cell proliferation, apoptosis, and migration [12]. Besides, upregulation of miR-139-5p was reported to inhibit cell proliferation by directly targeting TPD52 in human uterus leiomyoma cells (UtLMCs) [13]. However, miRNAs have been shown to be correlated with the development and progression processes in many different cancers [14,15,16,17]. MiR-199a has been reported to be downregulated and represses tumor progression in prostate adenocarcinoma [14], chondrosarcoma [15], hepatocellular carcinoma [16], and ovarian cancer [17]. However, the effect and precise regulation mechanisms of miR-199a in ULM are still unknown. Besides, mediator complex subunit 12 (MED12) is an essential hub for transcriptional regulation, in which mutations and overexpression were reported to be associated with several kinds of malignancies [18]. Clinically, many articles have reported that *MED12* is an oncogenic gene that promotes the occurrence of uterine fibroids [19].

In this study, we investigated the expression pattern of microRNA-199a-5p (miR-199a-5p) and MED12 in both ULM tissues and cells. In addition, the results of functional assays demonstrated that miR-199a-5p inhibited the cell proliferation and induced apoptosis by directly targeting MED12 in ULM. These results may provide some clues for exploring new therapeutic targets for ULM treatment.

## 2 Materials and methods

### 2.1 Tissue collection

A total of 40 paired ULM tissues and corresponding tissues were obtained from patients in Zhangjiagang Hospital of Traditional Chinese Medicine who have not received radiotherapy or chemotherapy preoperatively. All tissue samples were stored at −80°C for further analysis.


**Ethical approval:** The research related to human use has been complied with all the relevant national regulations, institutional policies, and in accordance with the tenets of the Helsinki Declaration and has been approved by the Zhangjiagang Hospital of Traditional Chinese Medicine & Affiliated Zhangjiagang Hospital of Nanjing University of Chinese Medicine committee (2021NL-099-01).

### 2.2 Isolation and culture of UtLMC

The UtLMCs were isolated from small portions of leiomyoma and cultured by the digestion and tissue adherence methods as previously described [20]. To achieve convincing data, we isolated two lines of UtLMCs from different patients and defined them as UtLMCs-A and UtLMCs-B. All the cells were cultured in Dulbecco’s modified Eagle’s Medium (DMEM) containing 10% fetal bovine serum (Invitrogen, Carlsbad, USA) at 37°C in a humidified atmosphere with 5% CO_2_. Briefly, after being digested with trypsin and cultured for 24 h, the medium was replaced with fresh medium. Afterward, cells were examined per 24 h under a light microscope. When the cells reached confluence, cell passage was carried out. The experiment only included the cells within the sixth generation of the leiomyoma cells. Prior to use, the cell cultures were characterized using antibodies to α-smooth muscle actin (α-SMA) based on immunofluorescence microscopy. This cell culture was morphologically homogenous and at initial isolation and after first passage displayed 100% immunostaining for α-SMA.

### 2.3 Cell transfection

MiR-199a-5p mimics and the negative control (NC) were obtained from GenePharma (Shanghai, China). All the vectors of miRNAs were labeled with green fluorescence protein (GFP). Transfection was performed using Lipofectamine 2000 (Invitrogen, Shanghai, China) reagent according to the manufacturer’s instructions.

### 2.4 Real-time quantitative PCR (qPCR)

Total RNA was extracted from tumor tissues or cells using Trizol reagent according to the manufacturer’s protocol. The RNA expression levels of miR-199a-5p, MED12, and β-actin were amplified by qPCR using the SybrGreen reagent (Takara, Dalian, China) on a Strata gene 3005p system (Agilent Technologies, Mississauga, Canada). The housekeeping genes, including β-actin, were used as the internal standards, respectively. A 2^−ΔΔCT^ method was used to measure the relative expression level. Primer sequences were designed as follows: miR-199a-5p forward, 5′-GCTTTGGCATCACCCCTAAT-3′, reverse, 5′-CCCAGTGTTCAGACTACCTGTTC-3′; MED12 forward, 5′-GCCCTTTCACCTTGTTCCTT-3′, reverse, 5′-TGTCCCTATAAGTCTTCCCAACC-3′; β-actin forward, 5′-CACCATTGGCAATGAGCGGTTC-3′, reverse, 5′-AGGTCTTTGCGGATGTCCACGT-3′; and U6 forward, 5′-CTCGCTTCGGCAGCACA-3′, reverse, 5′- AACGCTTCACGAATTTGCGT-3′.

### 2.5 Cell counting kit 8 (CCK-8) assay

The CCK-8 assay was used to determine the cell growth of UtLMCs. The cells were plated in 96-well plates at 5 × 10^3^ cells per well and cultured for 24, 48, and 72 h, respectively. Subsequently, CCK-8 solution was added each well for additional 2 h at 37°C. The absorbance was measured at 450 nm using a microplate Reader (Bio-Rad, Shanghai, China).

### 2.6 Colony formation assay

Transfected cells (5 × 10^3^ cells/well) were seeded in six-well plates and cultured in DMEM. Then, the medium was replaced every 3 days. Two weeks later, after a 12 day incubation, these cells were fixed with paraformaldehyde for 30 min, and stained with 10% crystal violet for another 30 min. Colonies were counted and photographed with a light microscope (Olympus, Tokyo, Japan).

### 2.7 5-Ethynyl-20-deoxyuridine (EdU) assay

For EdU assay, cells were incubated for 48 h at 37°C with 5% CO_2_, 100 μL of EdU (50 μM) was added into each well and cultured for 8 h. Cells were fixed with paraformaldehyde for 20 min, and Triton X-100 was used to permeabilize the nuclear membrane. Finally, cells were stained according to the manufacturer’s suggestions (Life Technologies, New York, USA).

### 2.8 Flow cytometry assay

The role of miR-199a-5p in apoptosis of UtLMCs was assessed using flow cytometry analysis. In brief, UtLMCs (1 × 10^4^ cells per well) were seeded in 24-well plates. Subsequently, 300 μL of 1× binding buffer was added in each well, and the treated cells were washed with phosphate-buffered saline twice. After that, 5 µL of annexin V-fluorescein isothiocyanate and 5 µL of propidium iodide were added for 15 min at room temperature. Finally, apoptosis cells were detected through a FACScalibur flow cytometer (BD Biosciences, CA, USA).

### 2.9 Western blot analysis

Proteins from tumor tissues or cells were extracted according to the manufacturer’s protocol, and the protein concentrations were determined by using bicinchoninic acid assay protein assay kit (Beyotime, Beijing, China). An equal number of proteins were loaded on 10% sodium dodecyl sulfate polyacrylamide gel electrophoresis for electrophoresis separation. The protein bands were transferred to polyvinylidene difluoride membranes and then incubated at 4°C overnight with primary antibodies. After that, the protein bands were incubated with horseradish peroxidase-conjugated secondary antibody at room temperature for 1 h. The protein signal was detected using enhanced chemiluminescence detection reagents (Thermo Scientific, CA, USA), and glyceraldehyde 3-phosphate dehydrogenase was used as the internal reference.

### 2.10 Luciferase targeting assay

The putative binding site in 3'-UTR of MED12 was mutated using mutagenesis kit. Wild type (WT) and mutant (Mut) sequences were amplified and inserted into the vector to construct luciferase reporter plasmids according to the manufacturer’s recommendations (Promega, Madison, USA). The luciferase activities were detected with the dual luciferase reporter kit (Promega, Madison, USA). For transfection, 200 ng of WT or Mut reporter vectors were transfected into UtLMCs in combinations with miR-199a-5p mimics. Relative luciferase activities (ratios of renilla luciferase signal normalized to firefly luciferase) were determined at 48 h time point after transfection.

### 2.11 Statistical analysis

Statistical product and service solutions (SPSS) (18.0 version, SPSS, Inc., USA) and GraphPad (6.0 version) were used to determine statistical data for each group. All data were presented as mean ± SD. All experiments were independently implemented at least three times. Moreover, Student’s *t*-test and one-way analysis of variance were used to compare two or more than two groups. *P* <0.05 indicated that the difference was statistically significant.

## 3 Results

### 3.1 MiR-199a-5p is downregulated in ULM tissues

**Figure 1:**
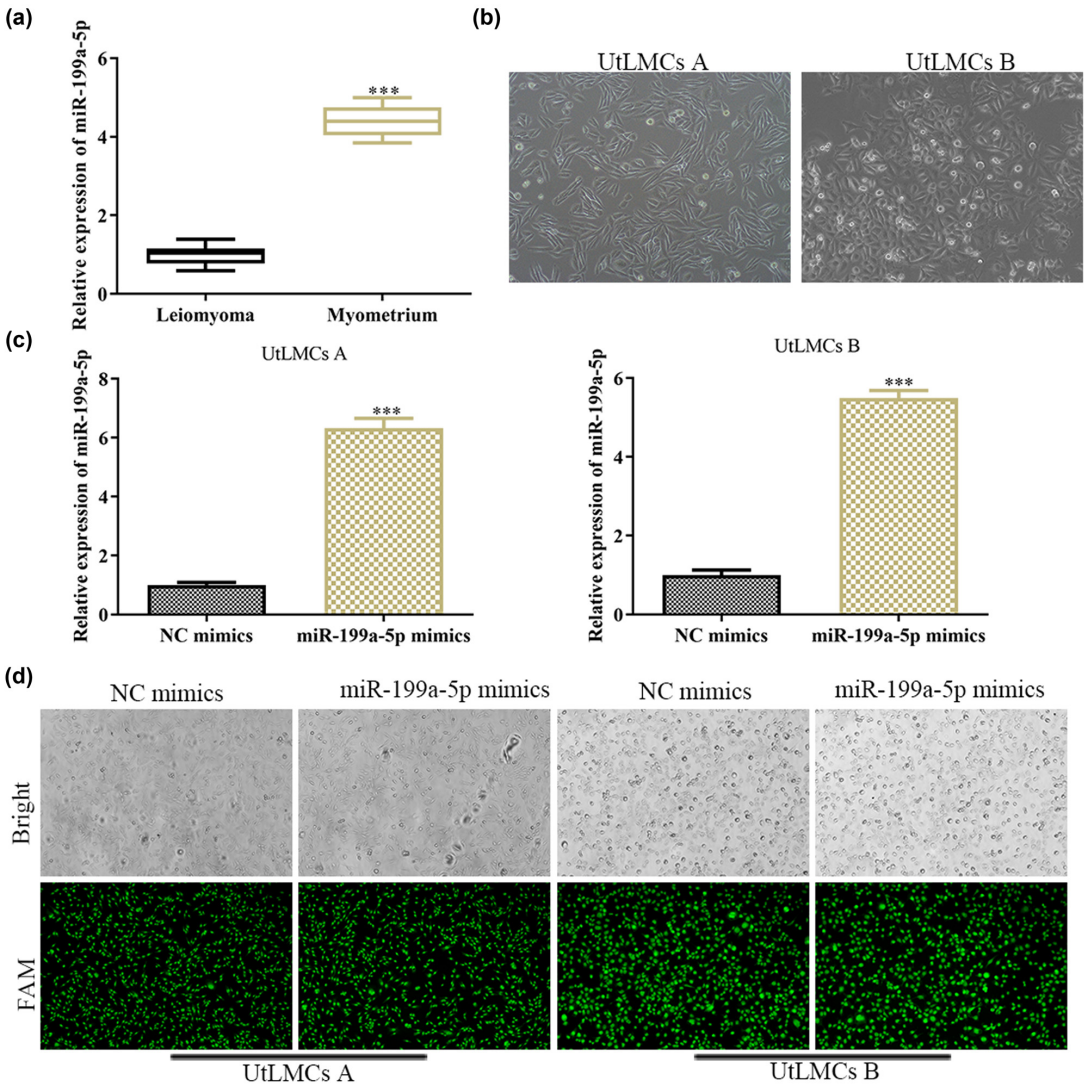
MiR-199a-5p is downregulated in ULM. (a) Relative expression level of miR-199a-5p in 40 paired ULM tissues and adjacent normal myometrium tissues. (b) Comparison of the morphological characteristics of human UtLMCs. (c) qPCR analysis of expression level of miR-199a-5p and (d) GFP fluorescence analysis of the transfection efficiency of miR-199a-5p mimics. All values are presented as mean ± SD from at least three separate experiments. ^***^ *P* <0.001 vs NC mimics group.

To explore the expression levels of miR-199a-5p in ULM, qPCR analysis was performed to assess the expression of miR-199a-5p in ULM tissues. As shown in Figure 1a, qPCR analysis revealed that the expression level of miR-199a-5p was decreased in ULM tissues, when compared with that in the paired adjacent normal myometrium tissues. After that, we found that when the density of cell reached 80% of the plate, the shape of the cells appeared spindle-shaped with dominant oviform nuclei. Moreover, we compared the morphology of the two cell lines from different tissues and found that they were in similar phenotypes (Figure 1b). After transfection of NC mimics and miR-199a-5p mimics, qPCR was carried out to detect the expression level of miR-199a-5p mimics. NC mimics and miR-199a-5p mimics were labeled with 6-fluorescein amidite, so the inverted fluorescence microscope was used to validate the transfection efficiency of miR-199a-5p mimics (Figure 1c and d). The results showed that miR-199a-5p is upregulated in ULM.

### 3.2 MiR-199a-5p inhibits cell proliferation and induces apoptosis in UtLMCs

**Figure 2:**
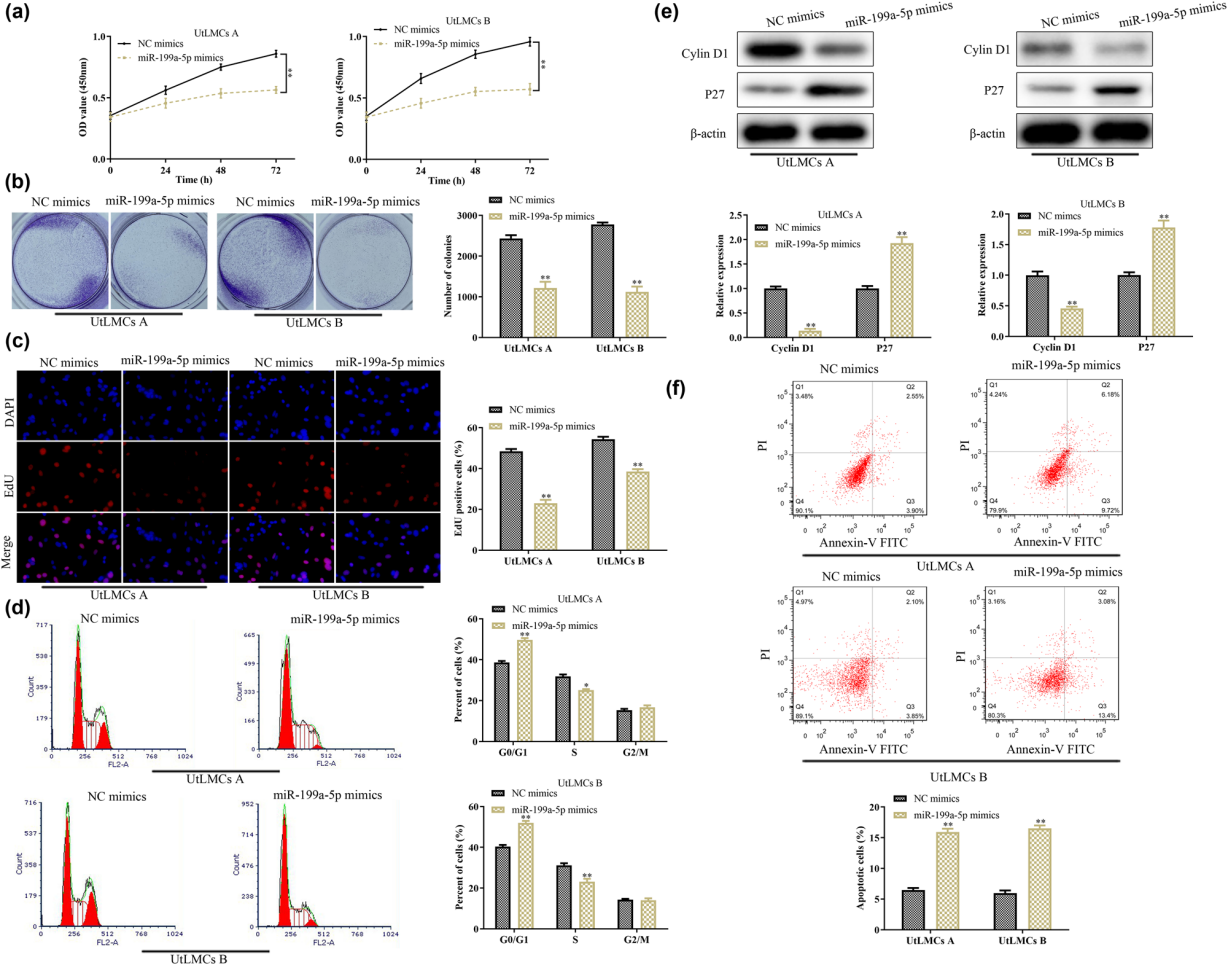
MiR-199a-5p inhibits cell proliferation and induces apoptosis of UtLMCs. The (a) CCK-8, (b) colony formation, and (c) EdU assays were used to assess cell proliferation. Flow cytometry was used to detect the effect of miR-199a-5p on (d) UtLMC cycle and (f) cell apoptosis. (e) The protein expression levels of cyclin D1 and P27 in UtLMCs were detected by Western blot. All values are presented as mean ± SD from at least three separate experiments. ^*^ *P* <0.05 and ^**^ *P* <0.01 vs NC mimics group.

Given the reduction of miR-199a-5p in ULM, we overexpressed the miR-199a-5p in the UtLMCs. As shown in Figure 2a, miR-199a-5p mimics significantly inhibited the proliferation of UtLMC-A and UtLMC-B as evidenced by CCK-8 analysis. These inhibitory effects were further confirmed by colony formation and EdU assays (Figure 2b and c). Given the importance of cell cycle involved in the regulation of the cell proliferation, we examined the effect of miR-199a-5p mimics on the UtLMC cycles. As shown in Figure 2d, we found that miR-199a-5p mimics arrested the cell cycle in G1 phase. At the molecular level, miR-199a-5p mimics remarkably decreased the expression levels of cell cycle-control proteins, cyclin D1, whereas increased the protein expression levels of cell cycle inhibitor P27 (Figure 2e). In addition, to further investigate the effect of miR-199a-5p on cell apoptosis, flow cytometry analysis was performed. The results indicated that the cell apoptosis rate was increased in the UtLMCs transfected with miR-199a-5p mimics (Figure 2f). These data suggested that miR-199a-5p functioned as an inhibitor for the UtLMC proliferation and an activator for the UtLMC apoptosis.

### 3.3 MED12 is a target of miR-199a-5p in UtLMCs

**Figure 3:**
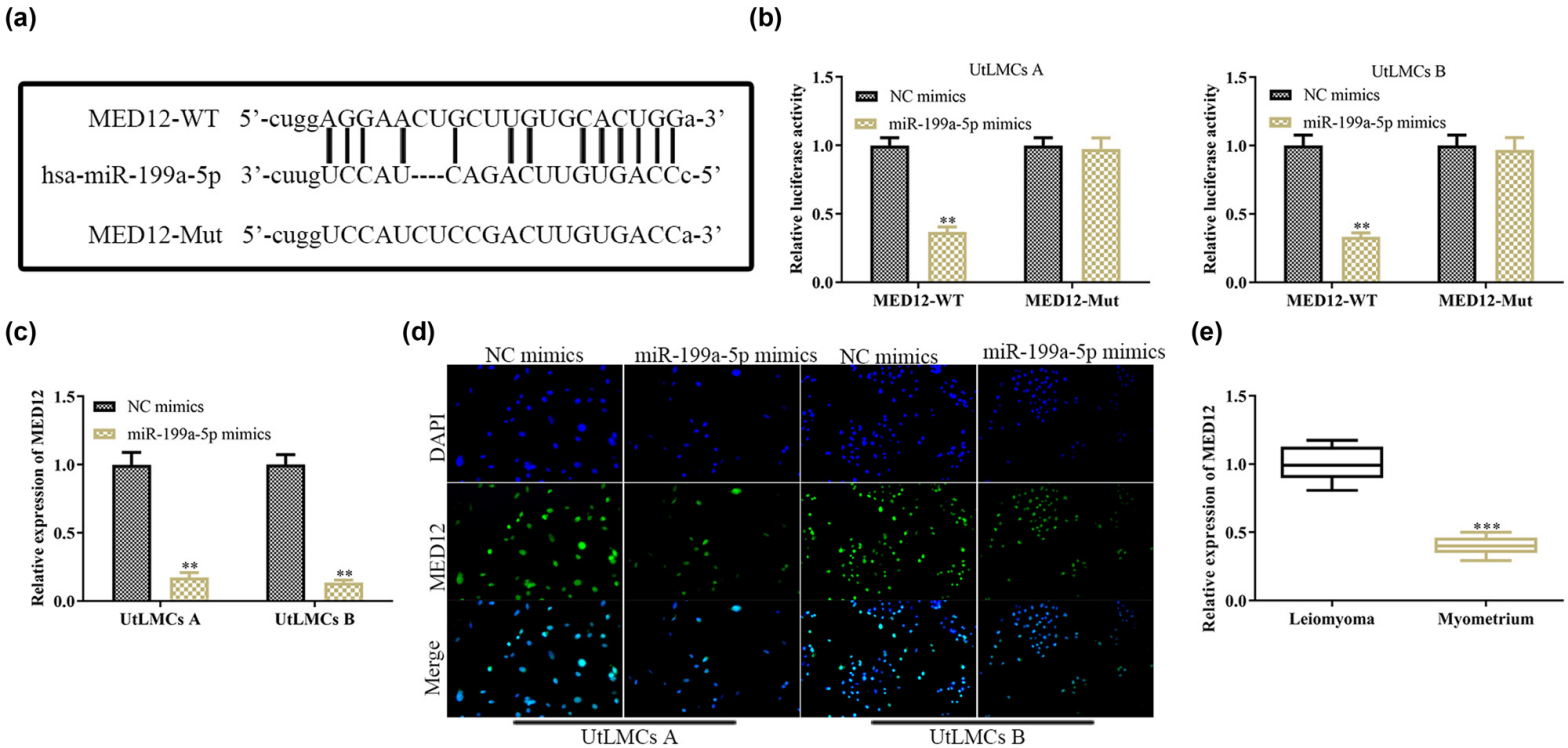
*MED12* is a target of miR-199a-5p in ULM. (a) Bioinformatics study showed that 3′-UTR of *MED12* contained a conserved binding site for miR-199a-5p. (b) Dual-luciferase reporter assay to validate the *MED12* as a target of miR-199a-5p. (c and d) qPCR and immunofluorescence analyses of mRNA and protein expression level of MED12 in response to miR-199a-5p overexpression. (e) Relative expression level of MED12 in 40 paired uterus leiomyoma tissues and adjacent normal myometrium tissues. All values are presented as mean ± SD from at least three separate experiments. ^**^ *P* <0.01 and ^***^ *P* <0.001 vs NC mimics group.

To explore the mechanism through which miR-199a-5p influenced the cell growth and apoptosis, we used bioinformatics tools to search for potential targets of miR-199a-5p. Among the predicted targets, MED12 was selected as the potential target of miR-199a-5p because of its oncogenic function. As shown in Figure 3a, StarBase analysis revealed that the 3′-UTR of MED12 contained a conserved binding site for miR-199a-5p (Figure 3a). To further consolidate that *MED12* was a target gene of miR-199a-5p, we transfected the UtLMCs with the miR-199a-5p mimics and NC mimics. We found that miR-199a-5p mimics dramatically decreased the luciferase activity of the 3′-UTR promoter of MED12, whereas this inhibitory effect was abolished when the putative binding sites were mutated (Figure 3b). Consistently, the expression levels of *MED12* were decreased at mRNA and protein levels (Figure 3c and d). In addition, the mRNA expression level of *MED12* was increased in the ULM tissues, when compared with that in the paired adjacent normal myometrium tissues (Figure 3e). Overall, these results validated that *MED12* was a direct target of miR-199a-5p.

### 3.4 MiR-199a-5p inhibits progression of ULM by targeting MED12

**Figure 4:**
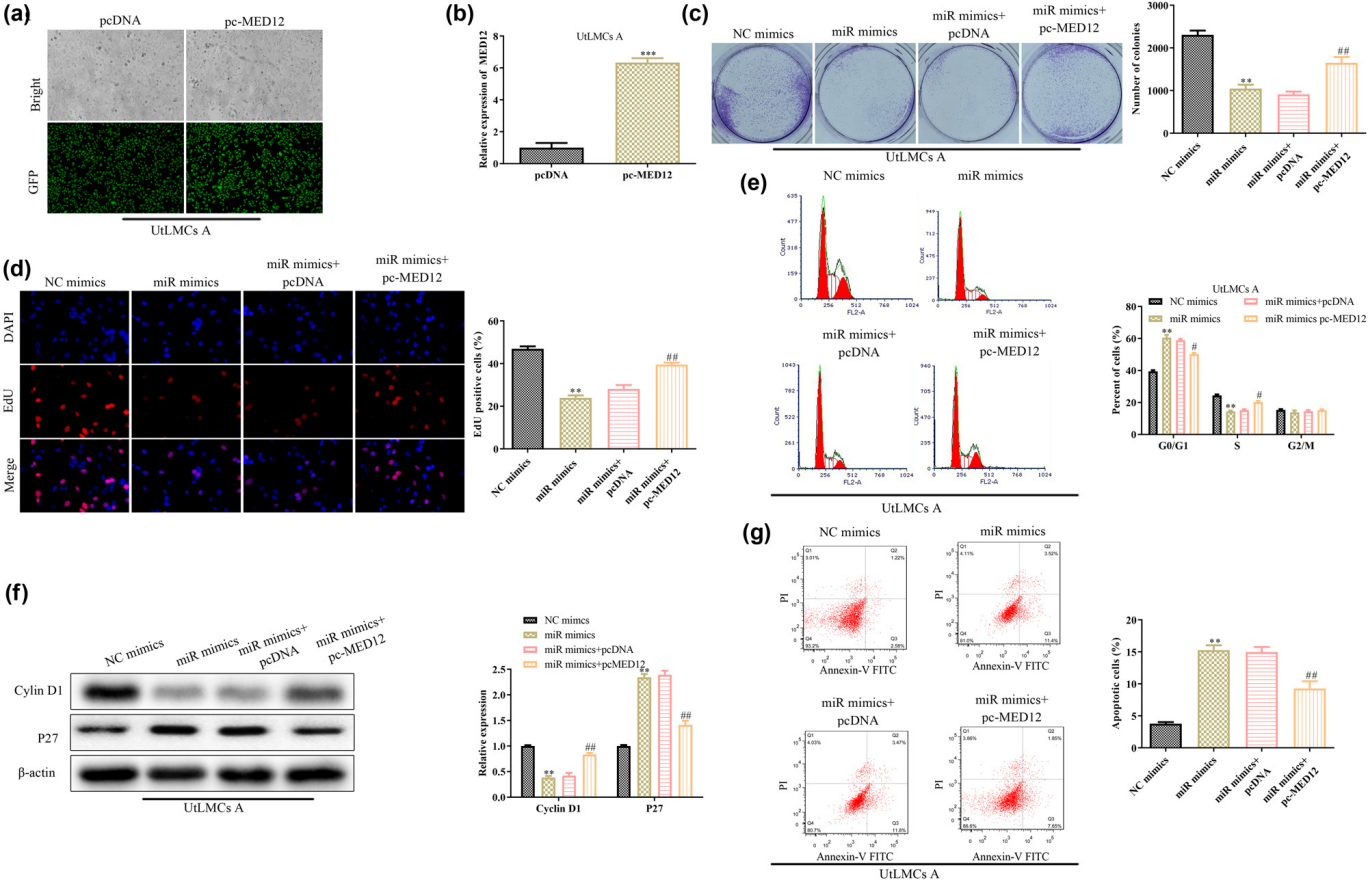
MiR-199a-5p inhibits progression of ULM by targeting MED12. Cells were transfected with miR-199a-5p mimics, pc-MED12 or their combinations. (a) qPCR and (b) GFP fluorescence analyses of the transfection efficiency of pc-MED. Cell proliferation was measured by (c) colony formation and (d) EdU assays. (e) Flow cytometry assay was used to assess the cell cycle. (f) The protein expression levels of cyclin D1 and P27 were detected using Western blot assay. (g) Flow cytometry assay was used to assess the cell apoptosis. All values are presented as mean ± SD from at least three separate experiments. ^**^ *P* <0.01 vs NC mimics group. ^#^ *P* <0.05, ^##^ *P* <0.01 vs miR-199a-5p mimics group.

To further demonstrate the role of MED12 in mediating the effects of miR-199a-5p on the ULM, we overexpressed the MED12 into the UtLMCs. qPCR and immunofluorescence analyses of mRNA and protein expression level of MED12 in response to miR-199a-5p overexpression are shown in Figure 4a and b. Functionally, MED12 overexpression partially restored the inhibitory effects of miR-199a-5p mimic on cell proliferation of UtLMCs (Figure 4c and d). At the molecular level, the expression of cyclin D1 was partially increased, whereas the P27 was decreased when the MED12 was overexpressed in the MiR-199a-5p-overexpressed UtLMCs (Figure 4e and f). However, flow cytometry assays indicated that the overexpression of MED12 prominently abrogated the miR-199a-5p mimic-induced promotion of cell apoptosis (Figure 4g). To this end, we have confirmed that *MED12* is indeed a molecular target and an output gene for miR-199a-5p in regulating the ULM progression.

## 4 Discussion

miRNAs have been shown to regulate gene expression level through degradation of mRNA and repression of its function. miRNAs play important roles in the regulation of cell proliferation, apoptosis, morphogenesis, and differentiation. Several miRNAs, including miR-197, miR-21, miR-23b, miR-29b, and let-7, and their predicted target genes are significantly dysregulated in ULM compared to normal myometrium. In recent years, researchers pay great attention and try to find an effective strategy to treat ULM. In this study, our results showed that the expression level of miR-199a-5p in leiomyoma was lower than that in the myometrium. MiR-199a-5p functioned as a potential antioncogene. The upregulation of miR-199a-5p in UtLMCs resulted in the inhibition of cell proliferation and arrest of cell cycle at G0/G1 phase. Additionally, we found that MED12 was *per se* a molecular target of miR-199a-5p and mediated the effects of miR-199a-5p on the proliferation and apoptosis of UtLMCs. As is known, MED12 plays an important role in cell growth and transformation of normal (myometrium) and tumorous smooth muscle cell tissues [21]. Thus, miR-199a-5p may function as an evolutionarily conserved antioncogene and a potential therapeutic target for ULM.

Differential expression levels of miR-199a-5p have been identified in several cancers with higher expression levels in prostate adenocarcinoma [14], chondrosarcoma [15], hepatocellular carcinoma [16], and ovarian cancer [17]. The level of miR-199a-5p is also found to be increased in the plasma of lung cancer patients with metastasis than in those without metastasis and significantly decreased in sensitive patients during chemotherapy [22–25]. In this study, we have examined the differential expression levels of miR-199a-5p in ULM and myometrium and found that the expression level of miR-199a-5p was decreased in ULM. Overexpression of miR-199a-5p in UtLMCs effectively suppressed cell growth.

In mammals, *MED12* is one of the most frequently mutated genes in breast fibroadenoma and is located on chromosome Xq13.1 [26]. It encodes for a protein that interacts with CDK8/CDK19, CYCC, and MED13 protein to form a mediator complex of RNA polymerase II, which participates in the regulation of transcriptional processes [27]. MED12 has been known to be frequently mutated in benign tumors, such as ULMs, phyllode, and prostate cancer. Highly recurrent somatic mutations in the *MED12* gene are reported in as many as 60–73% of breast fibroadenoma from Singapore [28]. The high prevalence of *MED12* mutations in fibroepithelial tumors suggests that it is a somatic driver gene for fibroepithelial tumorigenesis. Hotspot mutations of the *MED12* gene have been reported in exon 2 [28,29]. In our study, we found that the expression level of MED12 in ULM was significantly higher than that in the myometrium. Our data showed that the overexpression of *MED12* antagonized the regulatory functions of miR-199a-5p mimics in cell proliferation and apoptosis. Mechanistically, luciferase reporter studies showed that miR-199a-5p inhibited the expression level of *MED12* by directly targeting a conserved region on the 3′-UTR promoter of *MED12*. All these data suggested that *MED12* was a direct target of miR-199a-5p in ULM.

Previous similar studies have shown that the silencing of MED12 could reduce the proliferation of leiomyoma cells via the wingless-type (WNT)/β-catenin signaling pathway [30]. MED12 has been shown to interact with β-catenin physically and functionally, the key effector of canonical WNT signaling, to activate the transcription of target genes [31]. MED12 is also essential for the embryonic development, and its deletion disrupts both canonical WNT signaling and WNT/planar cell polarity [32]. It is highly possible that miR-199a-5p regulates the cell proliferation and apoptosis targeting at MED12 via the Wnt/β-catenin signaling pathway. We will focus on this aspect and carry out the further investigation in the future.

In summary, this study demonstrated that miR-199a-5p inhibited cell proliferation and induced apoptosis via targeting MED12 in ULM, which may provide a promising and novel molecular target for the treatment of ULM.
